# Transplantation of human amniotic epithelial cells promotes morphological and functional regeneration in a rat uterine scar model

**DOI:** 10.1186/s13287-021-02260-6

**Published:** 2021-03-24

**Authors:** Yihui Fan, Junyan Sun, Qiuwan Zhang, Dongmei Lai

**Affiliations:** 1 International Peace Maternity and Child Health Hospital, School of Medicine, Shanghai Jiao Tong University, Shanghai, 200030 China; 2 Shanghai Key Laboratory of Embryo Original Diseases, Shanghai, 200030 China; 3 Shanghai Key Clinical Speciality, Shanghai, 200030 China

**Keywords:** Human amniotic epithelial cells, Cesarean scar defect, Uterine scar, Matrix metalloproteinase-8, Fertility, Wound healing

## Abstract

**Background:**

Cesarean scar defect (CSD) is characterized by the presence of fibrotic tissue and decreased muscular density which is induced by cesarean section. Serious CSD may eventually result in infertility or obstetrical complications. Human amniotic epithelial cells (hAECs) have shown great promise in tissue regeneration. This study aims to investigate whether hAEC transplantation has the therapeutic effects on the rat uterine scar following full-thickness injury.

**Methods:**

A rat uterine scar model was established by excising the full-thickness uterine wall of about 1.0 cm in length and 1/2–2/3 of the total circumference in width. At day 30 post-surgery, hAECs were transplanted into the uterine scar. At day 30 and 60 post-transplantation, hematoxylin and eosin (H&E) staining, Masson staining, and IHC staining for vWF, VEGFA, α-SMA, and MMP-8 were performed to evaluate the regeneration of the scarred uterus and the underlying mechanism. Pregnancy outcomes were assessed at day 60 after hAEC transplantation. Finally, hAECs were incubated with hydrogen peroxide to verify the paracrine effect of hAECs.

**Results:**

Collagen deposition, thin myometrium, and injured endometrium were observed in the rat uterine scar model. After hAEC transplantation, collagen deposition in the uterine scar decreased, and myometrial and endometrial recovery was facilitated. hAEC transplantation also increased the fetus number implanted within the scarred area. Moreover, we found hAECs promoted angiogenesis via upregulation of VEGFA and decreased collagen deposition by upregulating MMP-8 in the uterine scar. The in vitro studies further demonstrated an increase in the expression level of MMP-8 in hAECs cultured with hydrogen peroxide.

**Conclusions:**

These results suggested that hAEC transplantation may be efficacious in the functional repair and collagen degradation of uterine scars, which provides a new therapeutic strategy to CSD.

## Background

The rate of cesarean section (CS) delivery has rapidly increased worldwide in the past few decades. In 2015, the frequency of CS was about 21.1% globally, which was twice of that in 2000 (12.1%), especially in Brazil and China [1]. In China, the overall CS rate decreased from 49.0 to 40.6% from 2011 to 2016 [2]. However, the frequency of CS remains much higher than 15%, the optimal frequency recommended by WHO. Recently, the long-term complication of CS, cesarean scar defect (CSD), has been of great concern. CSD, also called isthmocele or niche, is defined as discontinuation of the myometrium in the uterine scar of the cesarean section due to defective healing [3, 4]. The pathological features of CSD are the presence of fibrotic tissue and significantly decreased muscular density in the myometrium covering the scar [5]. The prevalence of CSD was approximately 45.6–64.5% worldwide and kept rising as the number of cesarean deliveries increased [6–8]. CSD could trigger infertility and obstetric complications in subsequent pregnancies like scar pregnancy, placenta accrete, placenta previa, and uterine rupture [9]. In addition, current therapies for CSD are limited. For patients with severe symptoms including infertility, surgery could be the only recommended therapy, including hysteroscopic, laparoscopic, and transvaginal surgical repair. However, there is no consensus on the optimal treatment of CSD [10, 11]. Consequently, it is important to develop a novel therapy for CSD.

Stem cell therapy has been recognized as a promising treatment strategy for the regeneration of the uterus. Li et al. established a rat uterine scar model following full-thickness injury and then applied collagen scaffolds loaded with collagen-binding human fibroblast growth factor for treatment [12]. The same team also showed that umbilical cord-derived mesenchymal stem cells (UC-MSCs) on scaffolds facilitated collagen deposition and promoted regeneration of the endometrium, myometrium and blood vessels in the rat uterine scar model. In addition, stem cell transplantation has been reported to treat recurrent intrauterine adhesions of patients clinically [13].

Human amniotic epithelial cells (hAECs) derived from the placenta have been shown to have great potential to be a source of cell-based therapy in tissue regeneration for their safety, availability, and no ethical consideration. Experimental evidences show that hAECs exhibit antifibrotic effects in the liver fibrosis and lung fibrosis [14]. However, whether hAEC transplantation has the therapeutic effects on the uterine scars and the underlying mechanisms were unknown.

In the present study, hAECs were injected into rat uterine scars following full-thickness injury. Our study aimed to investigate the therapeutic effect of hAECs on a rat uterine scar model and to explore a novel, stem cell-based therapeutic strategy for CSD.

## Materials and methods

### Isolation and culture of hAECs

Human placentas were obtained from healthy women with informed consent under the protocol approved by the Institutional Ethics Committee of the International Peace Maternity and Child Health Hospital. hAECs were isolated and cultured as described previously [15]. In brief, the amniotic membranes were mechanically separated from the chorionic portion of the placenta, dissected into segments, and then digested with 0.25% trypsin/EDTA at 37 °C for 25 min. The resulting cell suspensions were seeded in 100-mm plates containing the complete growth medium consisting of Dulbecco’s modified Eagle’s medium (DMEM)/F12 (Gibco, Grand Island, NY, USA) supplemented with 10% fetal bovine serum (FBS, Gibco) and streptomycin (100 U/mL, Gibco) and incubated at 37 °C in a humid atmosphere consisting of 5% CO_2_. Once cells achieved 80–90% confluency, they were collected for the following experiments. hAECs used in the experiments had undergone fewer than two passages.

### Characterization of hAECs

#### Flow cytometry

For hAEC characterization, cells were harvested and incubated with labeled primary antibodies (CD146-PE: 361006, isotype control 400114; CD324-APC: 324108, isotype control 400122; SSEA4-FITC: 330410, isotype control 401317; HLADR-FITC: 11-9956-42; isotype control 11-4724-82; Biolegend, USA) at 4 °C for 30 min. Then the cells were washed twice with PBS and analyzed by a BD Accuri C6 (BD Biosciences, NJ, USA).

#### Immunofluorescence staining

hAECs were characterized for CK18 and vimentin by using the immunofluorescent labeling technology. Cells were fixed with 4% paraformaldehyde for 10 min, washed with PBS, permeabilized with 0.1% Triton X-100 for 30 min, and block with 1% BSA for 30 min. Cells were then incubated overnight at 4 °C with primary antibody (CK18: Boster Biological Technology, China; Vimentin: Santa Cruz, USA). After washing three times, cells were probed with secondary antibody conjugated with Alexa Fluor^@^ 488 and Alexa Fluor^@^ 594 (Thermo Fisher Scientific) simultaneously in the dark. Cells were counterstained with Hoechst (Beyotime Institute of Biotechnology, China) and observed under the fluorescence microscope (Leica, Wetzlar, Germany).

#### Cell tracking assay

To track the transplanted hAECs in uterine scars, hAECs were previously labeled with carboxyfluorescein succinimidyl ester (CFSE) according to the manufacturer’s instructions (CFDA SE Cell Proliferation and Cell Tracking Kit, Yeason). The rats transplanted with CFSE labeling hAECs were sacrificed at day 1, 2, and 3 post-transplantation. The uterine horns were then removed and embedded in optimal cutting temperature (OCT) compound (Sakura Finetek, Seattle, USA), and 5-μm fresh sections were generated. The slides were mounted with an antifade mounting medium with DAPI (Beyotime Institute of Biotechnology, China). Fluorescence images were obtained with a Leica DMI3000 microscope (Heidelberg, Germany).

### Animal experiments

All animal procedures were approved by the Institutional Animal Care and Use Committee of Shanghai and were performed in accordance with the National Research Council Guide for the Guide for the Care and Use of Laboratory Animals. Female Sprague-Dawley (SD) rats weighing between 250 and 300 g were obtained from Shanghai SLAC Laboratory Animal Co., Ltd. and were housed at the Department of Animal Experiments, Medical School of Shanghai Jiao Tong University. The rats were maintained in SPF conditions and allowed to adapt to the new environment for at least a week. Vaginal smears were obtained daily at 09:00 a.m. for estrous cycle studies, and only rats with consecutive 4-day estrous cycles were selected. Ninety-two uterine horns from 46 rats were randomly assigned to three groups, including the hAECs group (*n* = 32 uterine horns), PBS group (*n* = 32 uterine horns), and sham group (*n* = 28 uterine horns).

The rat uterine scar model was established at the diestrus stage as described previously [12, 16]. Briefly, after the rats were anesthetized by intraperitoneal injection of pentobarbital sodium (50 mg/kg), the uterine horns were exposed by an excision in the low midline abdomen. A segment of approximately 1.0 cm in length and 1/2–2/3 of the total circumference was excised from each uterine horn, and the mesometrium was retained (Fig. 2C). The four margins of the uterine defect were marked with a 6-0 nylon suture. After rinsing the abdominal cavity with saline, the rectus fascia and skin were sutured with a 4-0 silk suture in an interrupted fashion. But for the sham group, the uterine horn was left intact in the abdominal cavity without excision. The rats received an intramuscular injection of penicillin for 3 days postoperatively.

Thirty days after full-thickness excision of uterine walls, a second incision was made in the abdominal wall and two different transplant components were injected into four previously marked points surrounding each uterine scar respectively. For the hAECs group, 10^6^ hAECs in 50 μL PBS were injected into each uterine scar. Then 1 mL of 10^7^ hAECs was injected intraperitoneally the following day. For the PBS group, 50 μL PBS was injected into each uterine scar. The sham group did not undergo the second surgery. The rectus fascia and skin were sutured and the rats were raised for the following experiments (Fig. 2A, B). Besides, for cell tracking, 10^6^ CFSE-labeled hAECs in 50 μL PBS were injected into the four previously marked points around the uterine scar, and 50 μL PBS was injected as the negative control.

### Histological analysis

Uterine specimens were collected 30 days and 60 days after transplantation of hAECs. Specimens were fixed in 4% paraformaldehyde, dehydrated in graded alcohols, cleared in xylene, and finally embedded in paraffin. The embedded tissues were sectioned transversally at a thickness of 5 μm. Hematoxylin and eosin (H&E) staining was used to evaluate the morphological structure of the uterus. Masson staining was applied to observe the collagen deposition around the uterine scars according to the manufacturer’s instructions (60532ES74, Yeason, China).

### Immunohistochemical analysis

Sections were immunolabeled with anti-α-smooth muscle actin antibody (α-SMA, ab5694, Abcam, USA), anti-von Willebrand factor antibody (vWF, PB9273, Boster Biological Technology, China), anti-vascular endometrial growth factor A (VEGFA, ab1316, Abcam, USA), and anti-matrix metalloproteinase-8 antibody (MMP-8, 17874-1-AP, Proteintech, Chicago, USA), mouse anti-4-hydroxynonenal (4-HNE, ab48506, Abcam, USA), and mouse anti-8-hydroxyguanosine (8-OHdG, ab62623, Abcam, USA) which were diluted in 1% goat serum in PBS. The sections were incubated with 0.3% H_2_O_2_ in methanol to quench endogenous peroxidase activity. After that, the sections were treated with heated antigen retrieval solution containing sodium citrate solution or ethylene diamine tetraacetic acid (EDTA) for antigen recovery according to the instructions of specific primary antibodies. After being incubated with 10% goat serum to block nonspecific antibody binding sites, the sections were incubated with the primary antibodies at 4 °C overnight. Immunoreactivity was visualized using a Mouse and Rabbit Specific HRP/DAB Detection IHC kit (ab64264, Abcam, USA) according to the manufacturer’s instructions.

For evaluation of smooth muscle abundance, we measured the percentage of α-SMA-positive area (α-SMA-positive area of the injured region/total α-SMA-positive area) by Image-Pro Plus software (Media Cybernetics, Inc., Rockville, MD, USA). The blood vessel density was evaluated by counting microvascular vessels which were vWF positive from six randomly selected fields per section under a magnification of × 400. The levels of VEGFA, MMP-8, 4-HNE, and 8-OHdG were measured by the percentage of cells with positive signals from six randomly selected fields per section under a magnification of × 400.

### Cell Counting Kit-8 (CCK8) viability assay

Cell viability was measured using a CCK-8 detection kit (Yeason, China) according to the manufacturer’s protocol. hAECs were seeded into 96-well plates at a density of 1 × 10^4^ cells/well. After hAECs were treated with different concentrations of H_2_O_2_ (25, 50, 100, and 200 μM) for 12 h, 10 μL CCK-8 solution was added into each well. Then the cells were incubated at 37 °C for 2 h. Absorbance values were measured at the wavelength of 450 nm using a microplate reader (SpectraMax 190; Eppendorf, Hamburg, Germany) and experiments were repeated three times.

### Western blot analysis

The uterine specimens collected at day 5 post-transplantation and hAECs exposed to H_2_O_2_ were lysed in cold RIPA buffer (Yeason, China) supplemented with protease inhibitor cocktail (Yeason, China) and phosphatase inhibitor cocktail (Yeason, China). The protein concentrations were then quantified by a Pierce BCA Protein Assay Kit (23225, Thermo Fisher Scientific, USA). Proteins were separated in polyacrylamide gel and transferred to the PVDF membranes. Membranes were blocked with 5% non-fat milk in Tris-HCI buffer solution containing 0.1% Tween-20 (TBST) and separately incubated with anti-MMP-8 (17874-1-AP, Proteintech, Chicago, USA), anti-VEGFA (ab1316, Abcam, USA), and anti-β-tubulin (30303ES10, Yeason, China) diluted with 5% BSA in TBST at 4 °C overnight. After washing with TBST, membranes were incubated with HRP-conjugated anti-rabbit IgG (Yeason, China). The blots were visualized with an Enhanced Chemiluminescence Kit (Millipore, USA). The level of β-tubulin was used as the internal standard. The immunoreactive band intensities were quantified by ImageJ software (NIH, MD, USA).

### Enzyme-linked immunosorbent assay (ELISA)

For the measurement of MMP-8 in the culture medium of hAECs exposed to H_2_O_2_, a commercially available ELISA kit (Boster Biological Technology, China) was used according to the manufacturer’s instructions. The detection limits were 156 to 10,000 pg/mL. The supernatant derived from hAECs was filtered through a 0.22-μm filter, transferred to ultrafiltration conical tubes (Amicon Ultra-4 with membranes selective for 3 kDa), and centrifuged to concentrate the supernatant. The final concentration was adjusted to 10 times the concentration of the collected supernatant. All the samples and standards were measured in duplicate.

### Fertility test

Sixty days post-transplantation, female rats were mated with male SD rats whose fertility had been verified to assess the function of the scarred uterine horns. The female rats were sacrificed for uterine examination 16 days after the presence of vaginal plugs. The number, size, and implantation site of fetuses were recorded.

### Statistical analysis

We performed image acquisition and histological analysis blinded to the treatment allocation of each specimen. Histological data were presented as average ± standard deviation (SD). Student’s *t* test or an ordinary one-way analysis of variance (ANOVA) with Bonferroni correction for multiple comparisons was applied to analyze the data. The numbers of fetuses were presented as median and minimum and maximum. Mann-Whitney test or Kruskal-Wallis test was applied to analyze the fetus count data. The pregnancy rates and the implantation rates in the scarred uteruses were analyzed by the Fisher exact test. Significant differences were calculated using GraphPad Prism version 8 (GraphPad software, La Jolla, USA). Differences were considered statistically significant at *P* < 0.05.

## Results

### Characterization of hAECs

**Fig. 1:**
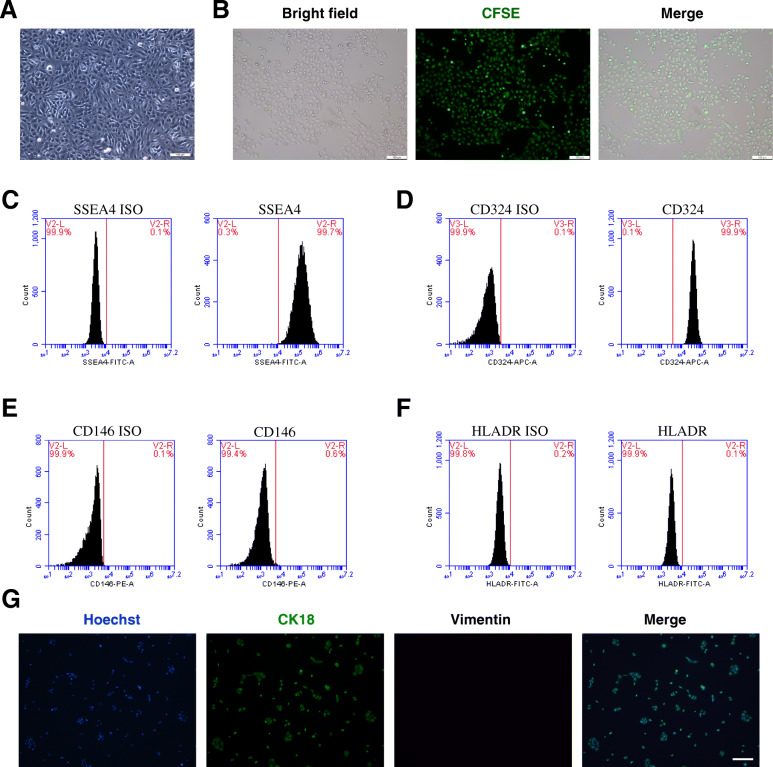
hAECs express specific surface markers and have stem cell characteristics with low immunogenicity. **a** hAECs presented an epithelial morphology under bright-field microscopy. Scale bar = 100 μm. **b** hAECs were labeled with CFSE to track implanted cells. The expression rate of green fluorescence staining was nearly 100%. Scale bar = 100 μm. **c-f **By flow cytometry, hAECs were positive for stem cell marker SSEA-4 (**c**) and epithelial marker CD324 (**d**) and were negative for mesenchymal markers CD146 (**e**) and HLA-DR (**f**). **g** Immunofluorescence staining for CK18 (an epithelial marker) expression and vimentin (a mesenchymal marker) in hAECs. Scale bar = 200 μm

Isolated hAECs cultured on a plastic dish exhibited typical cobblestone-like morphology (Fig. 1a). To identify the characteristics of hAECs, we performed flow cytometry and immunofluorescence staining. Flow cytometric analysis revealed that hAECs were positive for the stem cell marker SSEA4 (Fig. 1c) and the epithelial marker CD324 (Fig. 1d) while negative for the mesenchymal marker CD146 (Fig. 1e) and the immunogenicity indicator human leukocyte antigen HLA-DR (Fig. 1f). Immunofluorescence assays further showed that hAECs were positive for the epithelial marker CK18 and negative for the mesenchymal marker vimentin (Fig. 1g).

### Histological results

**Fig. 2:**
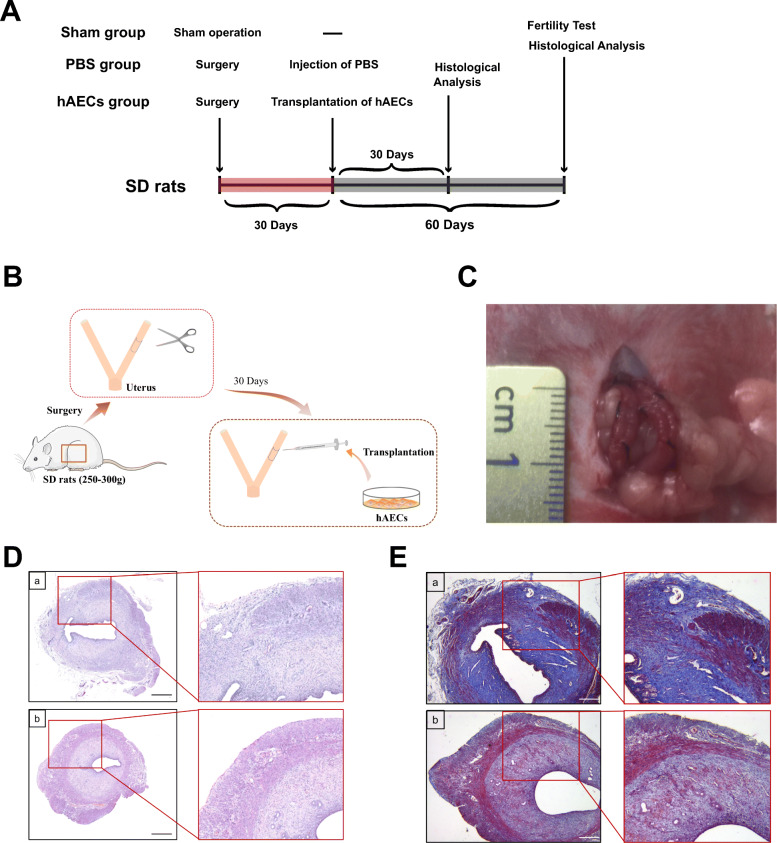
Schematic representation of experimental design and establishment of the animal model. **A** The timeline and design of the experimental flow of the uterine scar rat model. The red area of the timeline indicates different interventions and the gray area indicates the same procedures. **B** Diagram of establishment and treatment of the uterine scar rat model. A segment of around 1.0 cm in length and 0.5 cm in width (one-third to half of the uterine circumference) of the full-thick uterine wall was excised and removed while the mesometrium was retained. After 30 days, hAECs were transplanted into the uterine scar. **C** Gross image of the uterine scar rat model. **D** Hematoxylin and eosin (H&E)-stained cross-sections of the uterus in the uterine scar model group (a) and sham group (b). Scale bar = 500 μm. **E** Masson’s trichrome-stained cross-sections of uterine segments in the uterine scar model group (a) and sham group (b) 30 days after surgery. Scale bar = 200 μm. hAECs, human amniotic epithelial cells

We established a rat model for uterine scars following full-thickness injury. According to H&E staining and Masson’s trichrome staining, we observed that wounded uteruses had few endometrial glands and thin endometrium in the surgical region compared to the normal rat uterus at day 30 after injury (Fig. 2D). Masson’s trichrome staining showed that at day 30 post-injury myometrium was interrupted and collagen deposition was increased in the uterine scar model group compared to that in the sham group (Fig. 2E). Thus, these results validated the establishment of the rat uterine scar model.

**Fig. 3:**
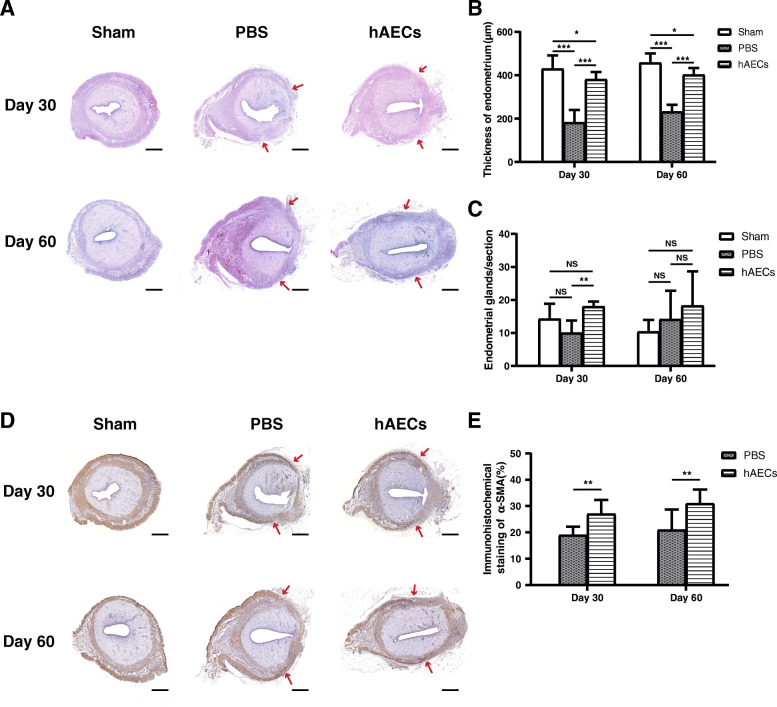
hAEC transplantation improved the recovery of endometrium and myometrium in scarred uteruses. **a** H&E staining of uterine scars at day 30 and 60 post-transplantation in the sham group, PBS group, and hAECs group (*n* = 8 uterine horns per group). Red arrows indicated repair sites. Scale bars = 500 μm. **b** Statistical analysis of the endometrial thickness in the uterine scars. **c** Statistical analysis of the number of endometrial glands per cross-section of the uterus. **d** Immunohistochemical (IHC) staining of α-smooth muscle actin (α-SMA) for smooth muscle abundance in uterine scars at day 30 and 60 post-transplantation in the sham group, PBS group, and hAECs group (*n* = 8 uterine horns per group). Red arrows indicated repair sites. Scale bars = 500 μm. **e** Statistical analysis of the percent of α-SMA-positive areas (α-SMA-positive area in the selected region/total α-SMA-positive area) measured by Image-Pro Plus software (*P < 0.05; **P < 0.01; ***P < 0.001; NS, *P* ≥ 0.05)

To investigate the effect of hAECs on the regeneration in rat uterine scars, hAECs were injected into the uterine scar using a microinjection needle at day 30 after injury. Then uterine specimens were collected at day 30 and day 60 post-transplantation separately. H&E staining was performed to assess endometrial thickness and the number of endometrial glands as shown in Fig. 3a. At day 30 post-transplantation, the thickness of the endometrium in the hAECs group was significantly higher than that in the PBS group (Fig. 3b). At day 60 post-transplantation, the thickness of the endometrium in the hAECs group (402.30 ± 30.97 μm) was also significantly higher than that in the PBS group (231.57 ± 32.83 μm), but lower than that in the sham group (457.68 ± 43.18 μm) (Fig. 3b). In addition, at day 30 post-transplantation, the number of glands per uterine cross-section in the hAECs group (18.00 ± 1.51) was significantly larger than that in the PBS group (10.00 ± 3.74) (Fig. 3c). However, there were no statistical differences in the number of glands between the hAECs group and the PBS group at day 60 post-transplantation (Fig. 3c).

**Fig. 4:**
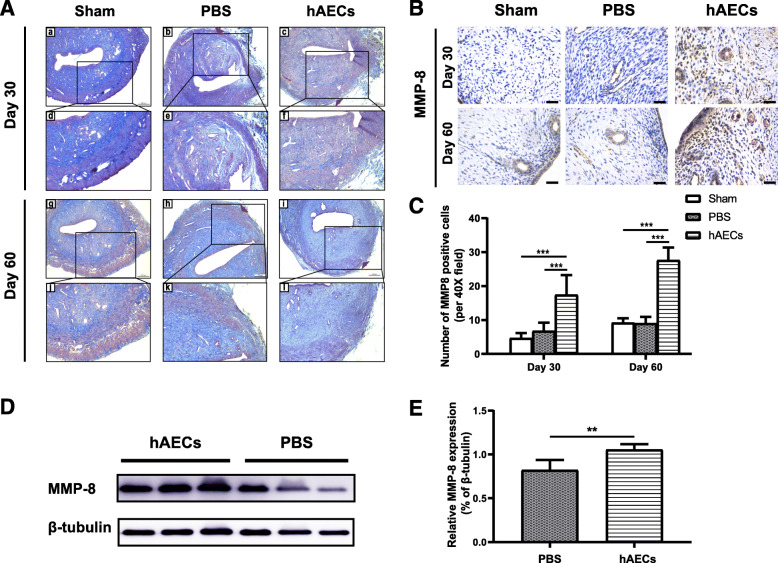
hAECs promoted the collagen degradation through increasing the expression of MMP-8. **A** Masson staining results showed less collagen deposition (stained blue) around the uterine scar in the hAECs group (i, l) compared with the PBS group (e, k). Scale bar = 200 μm. **B** IHC staining was used to detect the expression of MMP-8 in the uterine scars at day 30 and 60 post-transplantation in the sham group, PBS group, and hAECs group (*n* = 8 uterine horns per group). Scale bar = 25 μm. **C** The MMP-8 expression was semi-quantified by calculating the positive cells per field under a magnification of × 400. **D** Western blot analysis showed the MMP-8 expression of the uterine scars in the hAECs group and PBS group at day 5 after injection of hAECs or PBS. **E** The grayscale values of the western blots were evaluated. The protein level of MMP-8 was normalized to that of β-tubulin (*n* = 5; **P < 0.01; ***P < 0.001)

Collagen deposition is an essential feature of the uterine scar. Masson staining was used to evaluate the extent of collagen deposition in the uterine scar. At day 30 post-transplantation, the uterine scar in the hAECs group showed obvious collagen degradation and increased muscle bundles compared with that in the PBS group (Fig. 4A). At day 60 post-transplantation, the area of collagen deposition in the uterine scar in the hAECs group was smaller and the thickness of the myometrium increased, while the uterine scar in the PBS group remained collagen deposition and thin myometrium (Fig. 4A).

### Immunohistological analysis

The myometrium is another essential indicator for the regeneration of the uterine scar. At day 30 post-transplantation, the percentage of α-SMA-positive areas in the hAECs group (27% ± 9.2%) was significantly higher than that in the PBS group (19% ± 3.2%) (Fig. 3d, e). At day 60 post-transplantation, the percentage of α-SMA-positive areas in the hAECs group (31% ± 5.3%) was significantly higher than that in the PBS group (21% ± 7.7%), and even higher than that in the hAECs group at day 30 post-transplantation (Fig. 3d, e).

Matrix metalloproteinase-8 (MMP-8) is a member of the collagenase subfamily of matrix metalloproteinases (MMPs) [17]. MMP-8 has the ability to cleave interstitial collagens I, II, and III and participated in tissue remodeling processes of the uterus [18, 19]. Here we detected the expression of MMP-8 in the uterine scar in vivo. IHC staining results showed that at day 30 post-transplantation the number of MMP-8-positive cells in the hAECs group was significantly larger than that in the PBS group (Fig. 4B, C). At day 60 post-transplantation, the number of MMP-8-positive cells in the hAECs group (27.60 ± 3.78) was also larger than that in the PBS group (9.02 ± 1.94) (Fig. 4B, C).

**Fig. 5:**
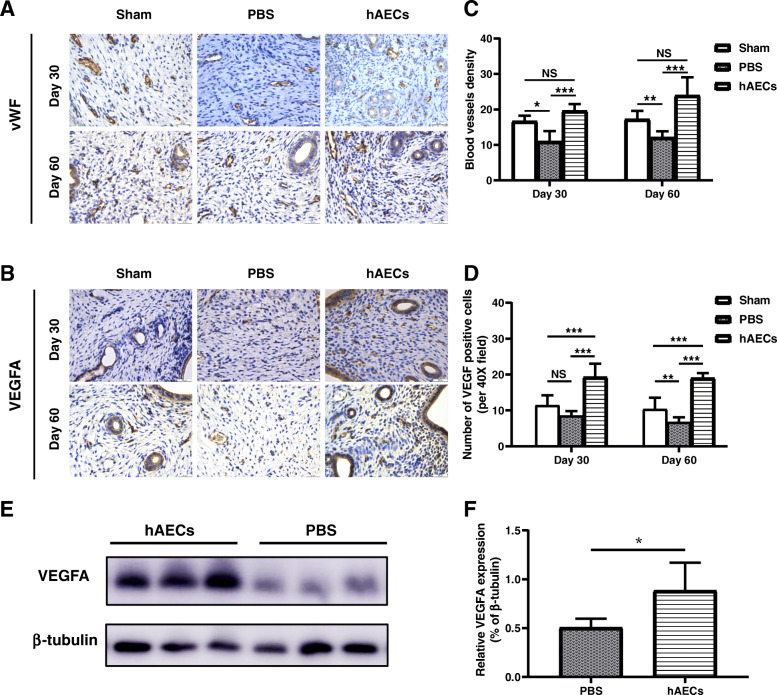
Effects of hAECs on angiogenesis in the injured uteruses. **a** IHC staining of von Willebrand factor (vWF) reflected the blood vessel density in the uterine scars of different groups at day 30 and 60 after injection of hAECs or PBS (*n* = 8 uterine horns per group). Scale bars = 25 μm. **b** IHC staining of VEGFA in the uterine scars at day 30 and 60 after injection of hAECs or PBS in different groups (*n* = 8 uterine horns per group). Scale bars = 25 μm. **c** Statistical analysis of the blood vessel density indicated by vWF-positive staining. **d** The VEGFA expression level was semi-qualified by calculating the percentage of positive cells per field under a magnification of × 400. **e** Western blot analysis showed that VEGFA expression of the uterine scars in the hAECs group and PBS group at day 5 after injection of hAECs or PBS. **f** The grayscale values of the western blots were analyzed. The protein level of VEGFA was normalized to that of β-tubulin (*n* = 5) (*P < 0.05; **P < 0.01; ***P < 0.001; ****P < 0.0001; NS, *P* ≥ 0.05)

Blood vessel regeneration plays an essential role in tissue repair. To study the effects of hAECs on vessel regeneration of the uterine scars, we performed the IHC staining for von Willebrand factor (vWF), a highly specific vascular endothelial marker. The results showed that at 30 days after transplantation the blood vessel density (BVD) of the hAECs group (19.60 ± 1.92) was significantly higher than that of the PBS group (10.44 ± 1.74) and was similar to that of the sham group (16.69 ± 1.53) (Fig. 5a, c). Consistent with this, at day 60 post-transplantation, BVD of the PBS group (12.13 ± 1.71) was lower than that of the sham group (17.25 ± 2.33), while BVD of the hAECs group (23.96 ± 5.11) was increased (Fig. 5a, c). To further explore the effects of hAECs on promoting angiogenesis, we conducted IHC staining for vascular endothelial growth factor A (VEGFA), a specific vascular endothelial cell growth-promoting factor. At day 30 post-transplantation, the number of VEGFA-positive cells in the hAECs group was significantly higher than that in the PBS group (Fig. 5b, d). At day 60 post-transplantation, the number of VEGF-positive cells in the hAECs group (19.00 ± 1.40) remained higher than that in the PBS group (10.35 ± 3.20) (Fig. 5b, d).

To determine whether oxidative damage increased in the uterine scar model group, we detected the expression of 4-HNE and 8-OHdG, which were widely accepted as biomarkers of oxidative damage productions [20], in the uterine scar in vivo (Additional file 2: Figure S2A,B). IHC staining results showed that oxidative lipid and DNA damage detected by 4-HNE and 8-OHdG respectively increased significantly in the uterine scar 30 days after injury compared to the sham group (Additional file 2: Figure S2C,D), indicating the oxidative stress damage at the injury site of uterine scar hAECs were implanted into.

### hAEC tracking in the uterine scar

**Fig. 6:**
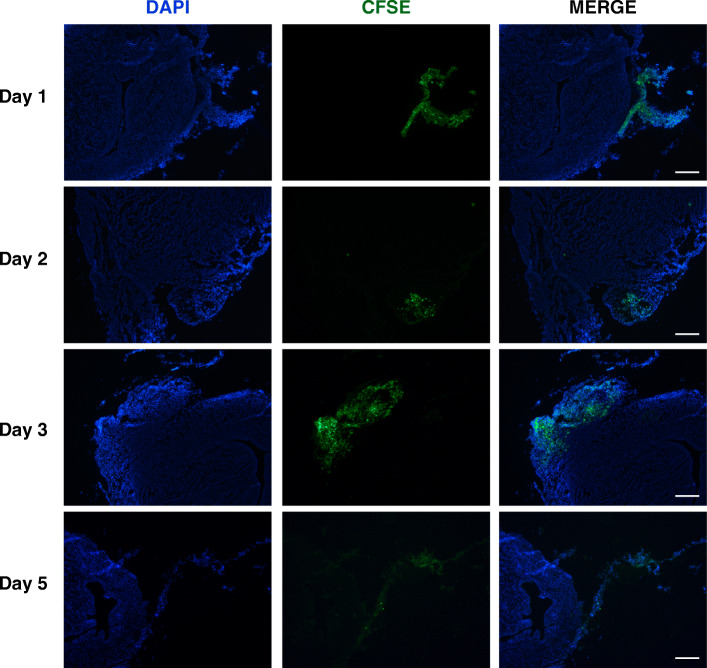
Localization of transplanted hAECs in the injured uteruses. CFSE-labeled hAECs were transplanted into uterine scars. At day 1, day 2, and day 3 after hAEC or PBS transplantation, uterine tissues containing the scarred areas were removed, embedded, and sectioned. Cell nuclei were stained with DAPI (blue). hAECs were tracked by the green fluorescence of CFSE under a fluorescence microscope. Scale bar = 200 μm

To determine whether hAECs were implanted into the uterine scar, we pretreated hAECs with CFSE which exhibited green fluorescence. Under the fluorescent microscope, we found CFSE-labeled hAECs surrounding the uterine scars at day 1, 2, and 3 after transplantation (Fig. 6). In the sections, hAECs were not located at the endometrium while they were near the outer layer of the injured uterine horn. In addition, immunofluorescence results indicated that hAECs in the uterine cells were positive for the epithelial marker CK18 and negative for the mesenchymal marker vimentin (Additional file 1: Figure S1).

### Cell viability analysis

**Fig. 7:**
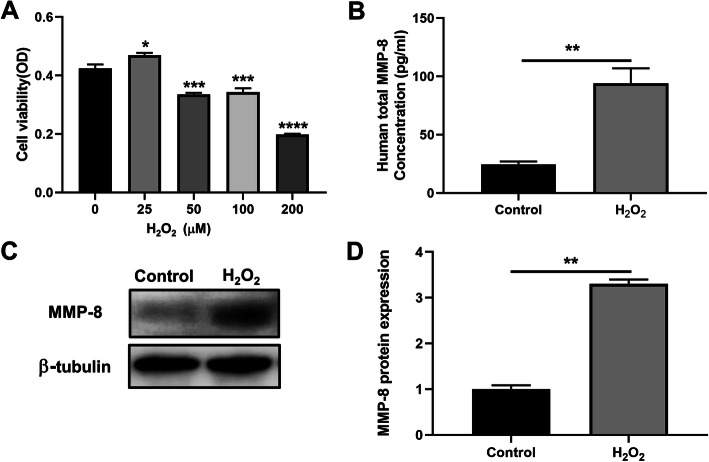
The expression levels of MMP-8 increased in hAECs treated with H_2_O_2_ in vitro. **a** The cell viability of hAECs treated with the indicated concentrations of H_2_O_2_ for 12 h by CCK-8 assay. The cell viability of hAECs increased after exposure to 25 μM H_2_O_2_ for 12 h; however, the viability of hAECs decreased significantly with the increase of H_2_O_2_ concentration (*n* = 3; *P < 0.05, ***P < 0.001, ****P < 0.0001 vs 0 μM H_2_O_2_). **b** Total MMP-8 levels in the supernatant derived from hAECs stimulated by H_2_O_2_ (25 μM) for 12 h were quantified by ELISA (*n* = 3; **P < 0.01). **c** The expressions of MMP-8 in hAECs exposed to H_2_O_2_ (25 μM) for 12 h were measured by western blot. **d** The relative expression level of MMP-8 normalized to β-tubulin (*n* = 3; **P < 0.01). Control, cultured with a normal medium; H_2_O_2_, cultured with a medium containing 25 μM H_2_O_2_

Hydrogen peroxide (H_2_O_2_) is considered as one of the important oxygen metabolites in redox signaling that composes the process of wound healing [21]. hAECs were cultured with a medium containing H_2_O_2_ to imitate the dysregulated environment after injury [22]. The CCK8 results showed that the cell viability of hAECs increased after exposure to 25 μM H_2_O_2_ for 12 h; however, the viability of hAECs decreased with the increase of H_2_O_2_ concentration (Fig. 7a).

### ELISA and western blot analysis

Here we investigated the changes in the MMP-8 levels in hAECs after exposure to H_2_O_2_ in vitro by ELISA and western blots. We treated hAECs cultured with 25 μM H_2_O_2_ as the H_2_O_2_ group and hAECs without H_2_O_2_ as the control group. Twelve hours later, the medium was changed with a fresh culture medium. The supernatant and hAECs were collected to evaluate the protein level of MMP-8 by ELISA and western blotting separately. Both results showed that the protein level of MMP-8 in the H_2_O_2_ group was significantly higher than that in the control group (Fig. 7b–d).

In addition, we examined the VEGFA level and MMP-8 level in the uterine scar at day 5 post-transplantation by western blotting. Results showed that the protein level of MMP-8 in the hAECs group was significantly higher than that in the PBS group (Fig. 4D, E). And the protein level of VEGFA in the hAECs group was significantly higher than that in the PBS group (Fig. 5e, f).

### Recovery of fertility in the uterine scar rats after hAEC transplantation

**Fig. 8:**
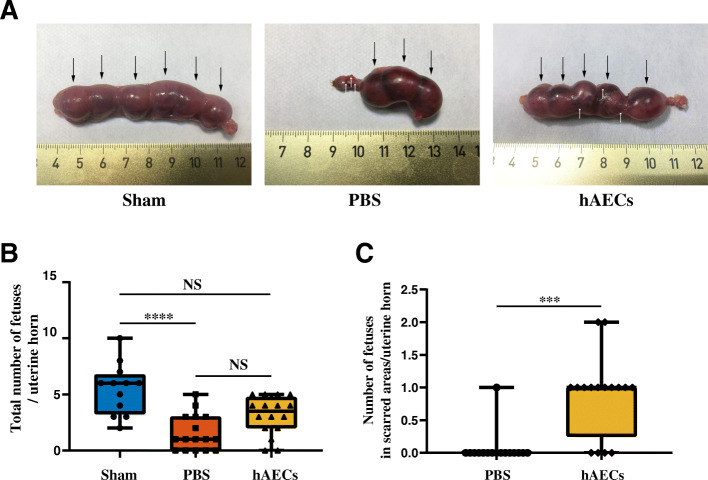
hAEC transplantation improved pregnancy outcomes in the uterine scar rat model. **a** Pregnancy outcome in uterine horns of female rats 90 days after injury in different groups. Similar size and shape of implanted fetuses were observed in the sham group; however, in the PBS group, there was no fetus implanted in the uterine scar. After hAEC transplantation, implanted fetuses were observed in the scarred area and were of similar size and shape to the fetuses implanted in the healthy area. Black arrows showed implanted fetuses and white arrows indicated the pre-marked margins of the uterine scar. **b** The total fetus number per uterine horn in the PBS group and hAECs group were smaller significantly than that in the sham group. **c** The number of fetuses implanted within the scarred area in the hAECs group was significantly larger than that in the PBS group. Data were presented as median, minimum, maximum, and individual data points (***P < 0.001; ****P < 0.0001; NS, *P* ≥ 0.05)

We further evaluated whether hAECs could improve pregnancy outcomes in the uterine scar rat model. Female rats were euthanized 16 days after the presence of the vaginal plug, and the uterine horns were analyzed whether they could maintain implanted fetuses up to the late viable stage of pregnancy. All rats in the sham group were pregnant (100%), while the pregnancy rate in the hAECs group (87.50%) was higher than that in the PBS group (68.75%) (Table 1). The total number of the fetuses per uterine horn in the hAECs group (3.50, range 0–5) and that in the PBS group (1.00, range 0–5) were lower than that in the sham group (6.00, range 2–10) (Fig. 8a, b). However, there were no statistical differences between the hAECs group and the PBS group. Further analysis of implantation sites demonstrated that the number of fetuses implanted within the scarred areas per uterine horn in the hAECs group (1.00, range 0–2) was significantly higher than the PBS group (0, range 0–1) (Fig. 8c).

**Table 1:** Reproductive outcomes among different treatments 90 days after injury

Variable	Sham group	PBS group	hAECs group	*P* value
Total number of uterine horns	12	16	16	
Pregnant uterine horns (%)	12 (100%)	11 (68.75%)	14 (87.5%)	0.0734
Uterine horns with fetus implantation in scarred areas (%)	–	1 (7.14%)	12 (75%)	0.0002

## Discussion

In the present study, we first evaluated the therapeutic effect of hAECs in a rat uterine scar defect model established by full-thickness injury. Here we found that hAEC transplantation improved the endometrial and myometrial regeneration and promoted collagen degradation, which contributed to the functional and morphologic recovery in the uterine scar, although there were no statistical differences between the number of glands in the hAECs group and that in the PBS group at day 60 post-transplantation as shown in Fig. 3c, which may be partially due to natural recovery 60 days after PBS injection. Moreover, hAEC transplantation restored the receptive fertility of the uterine scar.

Poor wound healing after surgery may result in scar formation where the damaged tissue can be replaced by the deposition of excessive extracellular matrix (ECM), ultimately causing tissue fibrosis and loss of organ function [23]. As a common medical approach for birth delivery, cesarean section may cause CSD, characterized by decreased myometrial thickness and excessive collagen deposition, inducing damaged uterine wall and impaired contractile properties of the uterus. Consequently, CSD may trigger infertility and serious obstetric complications in the future pregnancy. In our study, we established a rat uterine scar model which mimics the pathologic characteristics. By Masson staining, we observed excessive collagen deposition and low muscular density of the residual myometrium covering the scar in our model.

As one type of perinatal stem cells, hAECs isolated from discarded placenta have advantages of immune privilege, non-tumorigenicity, low cost, rich cell sources, and absence of ethical consideration, making them practical for broad clinical applications [24]. Recent studies have identified the paracrine effect of stem cells on the regeneration and repair of injured tissue. Stem cells could secrete trophic factors to alter the local microenvironment which play an important role in functional tissue repair [25]. It was reported that hAECs promoted the structural and functional regeneration of injured myocardial tissue through secreting pro-angiogenic cytokines including ANG, EGF, IL-6, and MCP-1 [26]. Matrix metalloproteinases (MMPs) participate in the process of wound healing including wound closure, inflammation, and tissue remodeling. Xu et al. investigated the therapeutic effect of umbilical cord-derived mesenchymal stem cells on the scaffolds in the uterine scars via upregulation of MMP-9 [16]. Our previous study showed that hAECs could secrete abundant proteins including MMP-8 according to a cytokine array on hAEC-derived serum-free conditioned medium [27]. Matrix metalloproteinase-8 (MMP-8) is shown as the most active against type I collagen which is the predominant component of fibrillar collagen. Recent studies demonstrated the important role of MMP-8 in the wound healing. A study reported that mice deficient in MMP-8 exhibited delayed wound healing and increased inflammation mediated by TGF-β1 signaling, supporting that MMP-8 could alleviate scarring [28]. Interestingly, local application of a selective MMP-8 inhibitor retarded wound healing while topical use of active recombinant MMP-8 promoted wound healing with the decreased inflammation and enhanced angiogenesis [29]. In addition to the function in the wound healing, MMP-8 also participates in the uterine physiological process. A previous study observed the high level of MMP-8 from 1 day to 5 days postpartum in the involuting uterus suggesting that MMP-8 might elicit an important role in the degradation of the collagen fibers during the uterine involution [18]. Consistent with these studies, our in vivo study found increased expression of MMP-8 in the hAEC-treated uterine scar by both western blotting and immunohistochemistry staining. Besides, immunostaining results indicated the oxidative stress damage (the increasing expressions of 4-HNE and 8-OHdG) in the uterine scar environment, and then we treated hAECs with H_2_O_2_ to mimic oxidative stress in vitro. Data from the in vitro studies showed that the protein expression level of MMP-8 was increased in both H_2_O_2_-treated hAECs and the supernatant derived from H_2_O_2_-treated hAECs. These results suggested that hAECs might promote the degradation of collagen through upregulating MMP-8, and part of increased MMP-8 levels might be secreted from hAECs.

Blood vessel growth plays an essential role in tissue repair, since vessels support cells at the wound site with nutrition and oxygen. Deficient local angiogenesis is considered a very likely contributor to the impair healing. Vascular endothelial growth factor (VEGF) is an important growth factor participating in new blood vessel formation during the tissue repair [23]. Lin et al. showed the repairing function of a collagen-binding VEGF in the remodeling of the scarred uterus [30]. Our previous studies demonstrated that hAECs had the pro-angiogenic effect through the tube formation assay in vitro [27, 31]. Here we found significantly increased blood vessel formation and high level of VEGFA in the hAEC-treated uterine scar, indicating that hAECs might promote the regeneration of uterine scars through therapeutic angiogenesis by upregulation of VEGFA.

Dysregulated inflammation is considered an important contributor to chronic, unhealing wound [32]. Recently, numerous studies have focused on the immunomodulatory properties of hAECs. It was demonstrated that hAECs could secrete many immunoregulatory factors like IL-6, IL-8, CXCL2, and TGF-β, which may recruit and activate neutrophils. As MMP-8 is also expressed at a high level in neutrophils, whether the increased MMP-8 level may also be secreted from neutrophils induced by hAECs remains to be further studied. Besides, future studies should investigate whether MMP-8 participates in the therapeutic angiogenesis combined with VEGF or regulated by VEGF.

## Conclusion

The present study demonstrated the efficient therapeutic role of hAECs in a rat uterine scar model. hAEC transplantation promoted the regeneration of endometrium, myometrium, and blood vessels in the uterine scar. hAECs may facilitate angiogenesis via upregulation of VEGFA, and collagen degradation via upregulating MMP-8 in the uterine scar. Moreover, hAEC transplantation improved the impaired fertility of the scarred uteruses. Taken together, hAEC transplantation may be a promising curative therapy for patients who suffer from uterine scar diseases such as CSD.

## Supplementary Information

**Additional file 1: Figure S1.** hAECs remained epithelial phenotype early after transplantation. Uterine scars were transplanted with CFSE-labeled hAECs. Uterine horns containing the scarred tissues were removed and sectioned at day 1, 2, and 3 post-transplantation. Then the sections were immunofluorescence stained with anti-CK18 and anti-vimentin antibody. Under a fluorescence microscope, the expression of CK18 was observed while the expression of vimentin was absent. Scale bar = 200 μm.

**Additional file 2: Figure S2.** Oxidative stress damage increased in the uterine scar rat model. **A** IHC staining of 4-HNE in the sham group (a, c) and uterine scar model group (b, d). **B** IHC staining of 8-OHdG in the sham group (a, c) and uterine scar model group (b, d). **C-D** 4-HNE and 8-OHdG expression levels were semi-quantified by calculating the percentage of positive cells per field under a magnification of × 400 (**P < 0.01; ***P < 0.001). Six uterine horns per group were used for experiments. a-b, scale bar = 200 μm; c-d, scale bar = 50 μm.

**Additional file 3: Table S1.** Primary antibodies used in this study.

## Data Availability

Not applicable.

## Abbreviations

CS: Cesarean section
CSD: Cesarean scar defect
H&E: Hematoxylin and eosin
hAECs: Human amniotic epithelial cells
MMP-8: Matrix metalloproteinase-8
SD: Sprague-Dawley
VEGFA: Vascular endothelial growth factor A
vWF: Von Willebrand factor
α-SMA: α-Smooth muscle actin
ECM: Extracellular matrix
4-HNE: 4-Hydroxynonenal
8-OHdG: 8-Hydroxyguanosine
