# Supplementary figures and images for: Transplantation of human amniotic epithelial cells promotes morphological and functional regeneration in a rat uterine scar model
Source: Stem Cell Res Ther. 2021 Mar 24;12:207. doi: 10.1186/s13287-021-02260-6 (PMC7992833; doi:10.1186/s13287-021-02260-6)

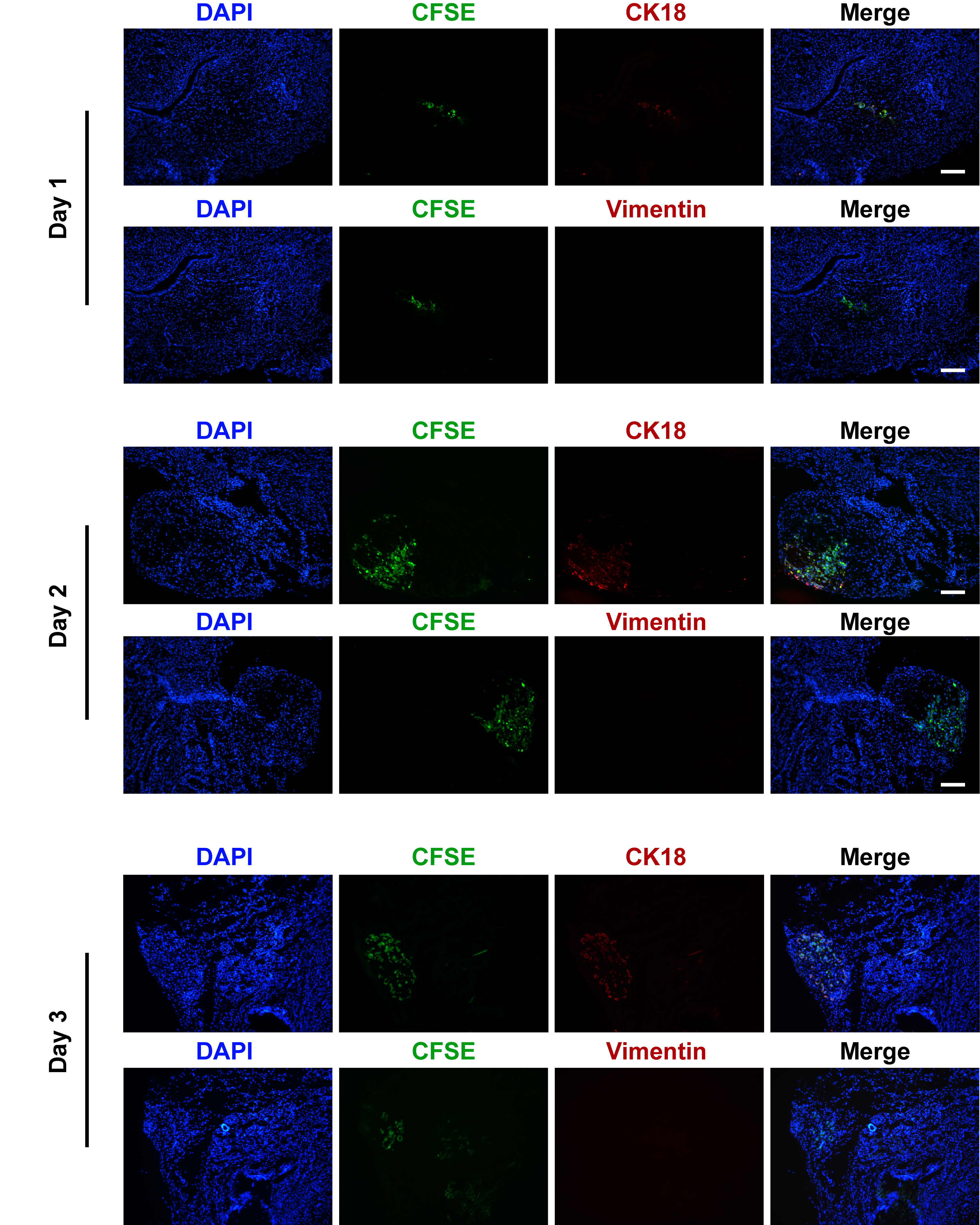

Supplement: Supplementary file 1 — Additional file 1: Figure S1. hAECs remained epithelial phenotype early after transplantation. Uterine scars were transplanted with CFSE-labeled hAECs. Uterine horns containing the scarred tissues were removed and sectioned at day 1, 2, and 3 post-transplantation. Then the sections were immunofluorescence stained with anti-CK18 and anti-vimentin antibody. Under a fluorescence microscope, the expression of CK18 was observed while the expression of vimentin was absent. Scale bar = 200 μm. [file 13287_2021_2260_MOESM1_ESM.tif]

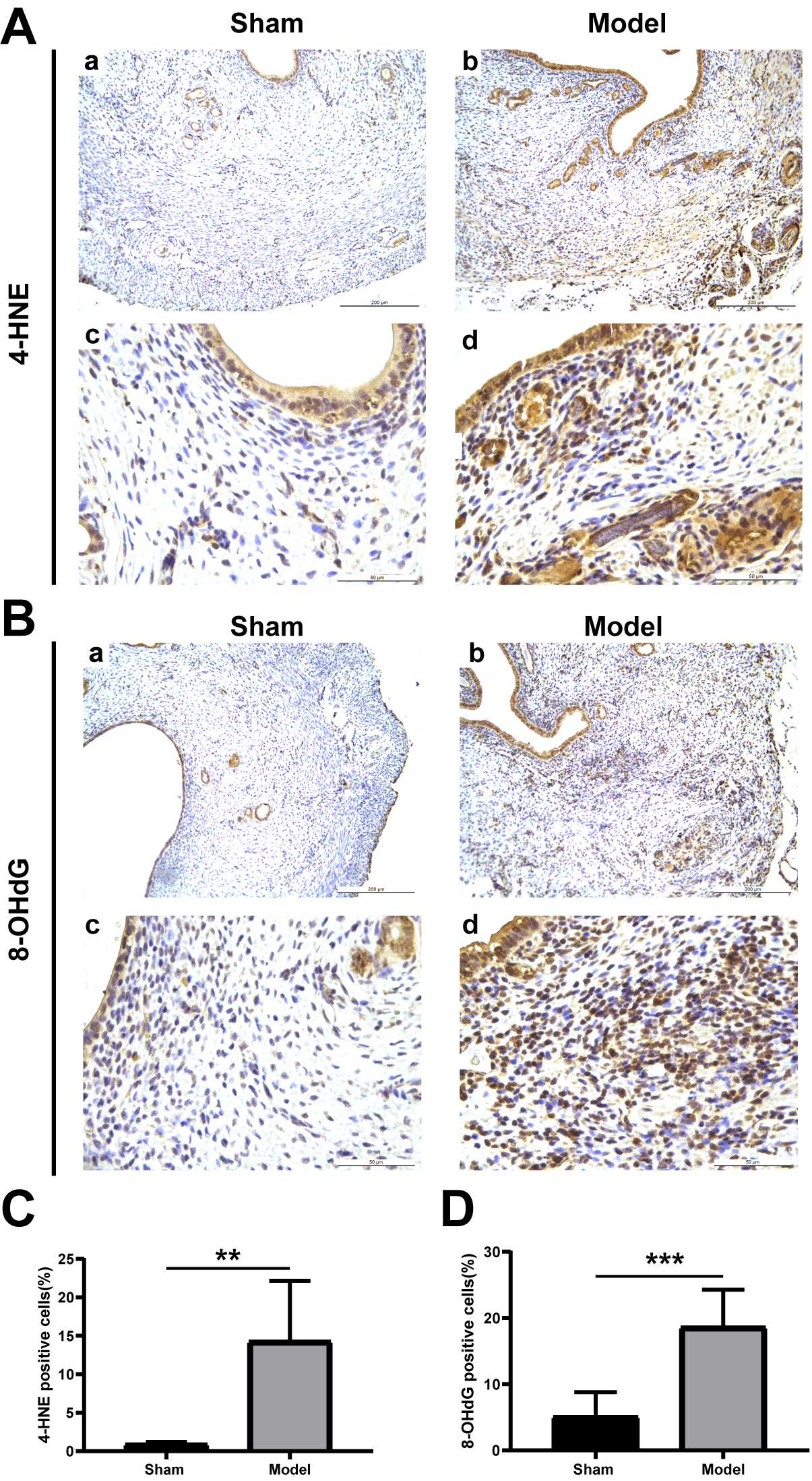

Supplement: Supplementary file 2 — Additional file 2: Figure S2. Oxidative stress damage increased in the uterine scar rat model. A IHC staining of 4-HNE in the sham group (a, c) and uterine scar model group (b, d). B IHC staining of 8-OHdG in the sham group (a, c) and uterine scar model group (b, d). C-D 4-HNE and 8-OHdG expression levels were semi-quantified by calculating the percentage of positive cells per field under a magnification of × 400 (**P < 0.01; ***P < 0.001). Six uterine horns per group were used for experiments. a-b, scale bar = 200 μm; c-d, scale bar = 50 μm. [file 13287_2021_2260_MOESM2_ESM.tif]
