# Supplementary material for: Transplantation of human amniotic epithelial cells promotes morphological and functional regeneration in a rat uterine scar model
Source: Stem Cell Res Ther. 2021 Mar 24;12:207. doi: 10.1186/s13287-021-02260-6 (PMC7992833; doi:10.1186/s13287-021-02260-6)
Supplement: Supplementary file 3 — Additional file 3: Table S1. Primary antibodies used in this study. [file 13287_2021_2260_MOESM3_ESM.docx]

**Additional file 3: Table S1 Primary antibodies used in this study.**

| Antibodies | Resource identifier | Catalog Number | Source |
| --- | --- | --- | --- |
| CD146-PE | AB_2562981 | 361006 | BioLegend |
| CD324-APC | AB_756070 | 324108 | BioLegend |
| SSEA4-FITC | AB_1089204 | 330410 | BioLegend |
| HLADR-FITC | AB_2572544 | 11-9956-42 | Thermo Fisher Scientific |
| CK-18 | —— | A01357-1 | Boster |
| Vimentin | AB_628436 | sc-32322 | Santa Cruz |
| α-SMA | AB_2223021 | ab5694 | Abcam |
| vWF | —— | PB9273 | Boster |
| VEGFA | AB_299738 | ab1316 | Abcam |
| MMP-8 | AB_2144582 | 17874-1-AP | Proteintech |
| 4-HNE | AB_867452 | ab48506 | Abcam |
| 8-OHdG | AB_940049 | ab62623 | Abcam |
